# Notification of Puerperal Fever

**Published:** 1900-09-22

**Authors:** 


### NOTIFICATION OF PUERPERAL FEVER.

It cannot be said that the discussion at Ipswich in regard to the notification of puerperal fever advanced matters very much, although in the end a resolution was passed to the effect that it was desirable that such notification should be generally adopted, which, of course, is already done wherever the Notification of Infectious Diseases Act has been put in force. In introducing the discussion, Dr. Berry Hart defined puerperal fever as an acute disease due to the entrance into the system from without, and through the genital tract, of a pathogenic organism, usually of a pyogenic character, but he showed some tendency to whittle down the all embracing nature of this definition by excluding sapræmic conditions and cases of localised lymphatic inflammations ending in pelvic cellulitis with or without suppuration, and also those cases in which the cause of the mischief was the gonococcus. This we think a pity, for we cannot but feel with Dr. Smyly and Dr. Boxall that, as a source of infection, local disease is as dangerous as a generalised attack. If the sanitary authorities propose to do anything to check the dissemination of puerperal fever, it is evident that what they want to be informed of is not merely the occurrence of very severe cases, regarding many of which, indeed, they would soon get information through the death certificates, but rather as to the prevalence of septic fevers of any degree in the practice of one or other doctor, nurse, or midwife, and this knowledge can only be obtained by all sorts of pyrexias after labour, except those of a merely evanescent or nervous type, being included in the definition. Much was made of the objections raised to notification on the ground that blame would be likely to attach in popular esteem to a medical practitioner in whose practice such cases occurred, and that the doctors would, in many cases, fail to notify except when cases were likely to terminate fatally; but we may doubt whether these objections are quite as real as some of the speakers seemed to imagine. At any rate, it was shown by Dr. Berry Hart that in large towns such as Sheffield, London, Leeds, &c., the notified cases considerably exceeded in number the fatal ones, which would suggest that where notification is in operation it is done conscientiously. It has so long been customary among our critics to blame the medical practitioner for these unfortunate cases, that people tend to forget that what a public official like the medical officer of health to a large district can do in searching out the origin of puerperal fever is by no means confined to tracking the medical attendant. There are many possibilities of infection in other directions. Both nurse and laundry come under suspicion, and it is only by notification that the matter can be traced back to its root. Dr. John Campbell, of Belfast, probably expressed the truth when he said that the nurse was responsible for a large proportion of the cases among the poor. Knowledge on the subject of antiseptics has become so widely spread throughout the whole body of the medical profession during the past quarter of a century, and the means by which a medical practitioner can free himself from infection are now so effectual, that even putting the matter on the lowest basis, namely, that of self-interest, it is inconceivable that medical practitioners should at all largely allow themselves to become the means of spreading puerperal fever among their patients. But the ordinary monthly nurse, who, so far as concerns the poor, is very often quite untrained in all that pertains to antiseptics, while even in regard to cleanliness her ideas are vague, remains, as she always has been, the probable source of much of the mischief. What we have to recognise is that, while puerperal fever has practically vanished from lying-in hospitals, the loss of life which it causes outside these hospitals has hardly diminished at all, and in seeking for the cause of this state of affairs it is only reasonable to attribute the continuance of this mortality to the continuance of the old custom of employing untrained women as nurses. We do not here refer to the midwife, but to the ordinary neighbourly nurse. The medical practitioner now possesses ample means of ridding himself of infection, and, so far as he himself is concerned, of putting a stop to the spread of this terrible disease, and in view of the fact that the disease still persists, we are forced to the conclusion that it has some other origin. The inquiry, then, must be carried further, and by aid of notification the medical officer of health must be put in a position in which he may trace any prevalence of puerperal sepsis to its origin, just as he would a milk outbreak of typhoid fever, and for this purpose the mild cases, as well as the serious ones, should be reported.

